# 

**Published:** 1849-10

**Authors:** 


### CONTENTS

### OF NO. IV.

---

### ORIGINAL COMMUNICATIONS.

### ESSAYS AND CASES.

Art. I.—Displacement of the Uterus, and some other diseases peculiar to females, with Cases. By R. L. Scruggs, M. D., of Germantown, Tennessee, - - - 277

Art. II.—Case of Extensive Fracture of the Skull, and Perforation of the Longitudinal Sinus—Operation and Recovery. By W. C. Cole, M. D., of Detroit, Mich. 286

Art. III.—A Case of Lienteric Diarrhea. By M. H. Fee, M. D., of Hiltonsville, Indiana, - - - 289

Art. IV.—Medical Heresies—Anesthesia in Natural Labor, etc. By Dr. E. B. Haskins, of Clarksville, Tennessee, 281

Art. V.—Wound of the Brain—Recovery. By William Kenney, M. D., of Millersburg, Ky. - - 307

Art. VI.—Treatment of Bloody Tumors by the Actual Cautery. Translated from the Gazette des Hôpitaux, by David W. Yandell, M. D., of Louisville, Ky. - 308

### REVIEWS.

Art. VII.—Manual of Physiology. By William Senhouse Kirkes, M. D. Assisted by James Paget, Lecturer on General Anatomy and Physiology, at St. Bartholomew's Hospital, - - - - - - 313

Art. VIII.—A Dissertation on the Practice of Medicine, containing an account of the causes, symptoms, and treatment of diseases: and adapted to the use of Physicians and Families. By Tomlinson Fort, M. D. - 334

Art. IX.—A Manual of Auscultation and Percussion. By M. Barth, Agrégé to the Faculty of Medicine at Paris, &c., &c., and M. Henry Roger, Physician to the Bureau Central of the Parisian Hospitals, &c., &c., &c. Translated, with additions, by Francis G. Smith, M. D., Lecturer on Physiology in the Philadelphia Association for Medical Instruction, one of the Editors of the Medical Examiner, &c., &c. - - - - - 336

SELECTIONS FROM AMERICAN AND FOREIGN JOURNALS.

Use of Nitrate of Silver for White Swellings, Hydrarthrosis, and Venereal Bubo, - - - - - - 337
On the Neck as a Medical Region, and on Paroxysmal Paralysis, - - - - - - - - 339
On the use of Glycerine in Diseases of the Ear, - - 348
Sulphate of Phyllerine, - - - - - - 360
Mesmerism in Holland, - - - - - - - 360

ORIGINAL INTELLIGENCE.

University of Louisville, - - - - - - 361
Death of Eminent Medical Men, - - - - 361
Subsidence of Cholera, - - - - - - 363
Fossil Elephant, - - - - - - 363
Southern Medical Reports, - - - - - - 364
Discovery of a New Cave in Kentucky, - - - - 364
New Work on Natural History, - - - - - 365
Prolonged Inanition, - - - - - - 365
Miss Blackwell, M. D. - - - - - - 366
Chloroform externally applied, - - - - - - 366
Memphis Medical College, - - - - - - 367
Transylvania Medical Journal. - - - - - 367
Quackery and the Legislature of Ohio, - - . 368
Annalist, - - - - - - - 368
New Orleans Medical Journal, - - - - - 368
Delinquencies, - - - - - - - 368

